# Adult-Onset Leukoencephalopathy with Axonal Spheroids and Pigmented Glia Caused by a Novel R782G Mutation in *CSF1R*

**DOI:** 10.1038/srep10042

**Published:** 2015-05-15

**Authors:** Nicola Foulds, Reuben J. Pengelly, Simon R. Hammans, James A. R. Nicoll, David W. Ellison, Adam Ditchfield, Sarah Beck, Sarah Ennis

**Affiliations:** 1 Wessex Clinical Genetics Services, University Hospital Southampton NHS Foundation Trust, UK; 2 Department of Human Genetics and Genomic Medicine, Faculty of Medicine, University of Southampton, UK. Wessex Neurological Centre, University Hospital Southampton NHS Foundation Trust, UK; 3 Clinical Neurosciences, Clinical and Experimental Sciences, Faculty of Medicine, University of Southampton, UK; 4 Department of Pathology, St. Jude Children’s Research Hospital, Memphis, USA; 5 Department of Radiology, University Hospital Southampton NHS Foundation Trust, UK

## Abstract

We report a new family with autosomal dominant inheritance of a late onset rapidly progressive leukodystrophy in which exome sequencing has revealed a novel mutation p.R782G in the Colony-Stimulating Factor 1 Receptor gene (*CSF1R*). Neuropathology of two affected family members showed cerebral white matter degeneration with axonal swellings and pigmented macrophages. The few recently reported families with *CSF1R* mutations had been previously labelled “hereditary diffuse leukencephalopathy with axonal spheroids” (HDLS) and “pigmentary orthochromatic leukodystrophy” (POLD), disorders which now appear to form a disease continuum. The term “adult-onset leukoencephalopathy with axonal spheroids and pigmented glia” (ALSP) has been proposed to encompass this spectrum. As CSF1R regulates microglia this mutation implies that dysregulation of microglia is the primary cause of the disease.

Adult onset leukodystrophies are a rare group of disorders with significant clinical and pathological heterogeneity. Leukodystrophies are characterised pathologically by extensive degenerative and /or demyelinating lesions in the cerebral white matter. Clinical manifestations can be very variable including behavioural or mood changes, dementia, Parkinsonism, spasticity, dystonia and seizures. Brain MRI changes may be helpful in some cases to determine the aetiology of the leukodystrophy, but are not always discriminatory. Until recently molecular characterisation of this group of disorders had remained elusive, but advances in genetic technologies have allowed significant progress to be made. The last few years has seen effective ante-mortem diagnosis in CADASIL (cerebral arteriopathy, autosomal dominant, with subcortical infarcts and leukencephalopathy)^1^,^2^ (Notch 3 mutations) and CARASIL (cerebral arteriopathy, autosomal recessive, with subcortical infarcts and leukencephalopathy)^3^,^4^ (HTRA1 mutations) and delineation of the autosomal dominant leukodystrophy associated with amplifications of the gene *LMNB1*^5^. Most recently, mutations in the Colony-Stimulating Factor 1 Receptor gene (*CSF1R)*, have been associated with causing hereditary diffuse leukencephalopathy with axonal spheroids (HDLS)^6^,^7^ and pigmentary orthochromatic leukodystrophy (POLD)^8^. Families reported so far with changes in this gene have presented with a mean age of onset in the fourth decade and an average disease course of six to nine years^6^,^9^. The condition has often presented with a frontal lobe syndrome including lack of social inhibition and poor judgement, and progressed to involve memory impairment, personality changes, motor impairment and ultimately seizures^6^,^7^,^9^. Neurological signs prominent in several reported individuals have been limb apraxia and early loss of speech. So far there have been no reported genotype-phenotype correlations with some families showing significant differences in disease presentation and course within different family members. Although the majority of families reported to date have shown a clear dominant family history together with a mutation that segregates appropriately, a recent publication has found a significant proportion of de-novo *CSF1R* mutations in their cohort^10^. It is possible that there has been ascertainment bias in families initially investigated for changes in this gene, with convincing dominant families constituting the majority. In addition most available data would suggest that mutations in *CSF1R* have a high penetrance for a particularly aggressive phenotype, but there is one reported instance of non-penetrance at age 69 years^10^. The family that we report was typical in that the initial clinical presentation was rather non-specific, but notable in that the disease course was particularly rapid in all three affected individuals.

CSF-1R is a cell-surface receptor for the cytokine macrophage colony-stimulating factor 1 (CSF-1), which is known to be involved in regulation of differentiation and proliferation of mononuclear phagocytic cells, including microglia in the central nervous system. The protein has an intracellular tyrosine kinase (TK) domain and an extracellular ligand-binding domain and all HDLS causing mutations have thus far been reported in the TK domain (exons 12 to 22). The mutation in the family we report is present in exon 18, *CSF1R*:p.R782G.

## Results

### Clinical Cases

1i presented at the age of 40 years with behavioural change and difficulties with her legs. The following year she had developed incontinence of urine and reduced fluency of speech and was admitted to hospital in status epilepticus. She underwent a brain biopsy which showed degenerative changes in the cerebral white matter as described below. An air encephalogram showed cerebellar and cerebral atrophy. 1i died a few weeks later, aged 41.

**Figure 1:**
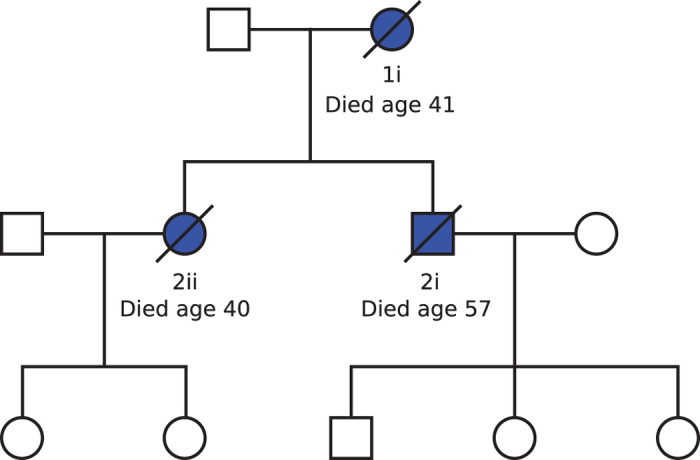
Family pedigree: Individuals shown in solid blue are those affected. A diagonal line indicates that the individual is now deceased.

2ii was the index case in this family (Fig. 1). She presented to clinical services at 38years with aching limbs. This was first investigated a year later following a fall which injured her left wrist and the pain in this arm failed to resolve and became a constant feature. At this time emotional lability and some memory loss had been noted by family. Her illness progressed rapidly to involve leg weakness and urinary incontinence and by the age of 39 years she was using a wheelchair. Aged 40 years she was admitted to hospital having had an episode of loss of consciousness and at this time was noted to have very slurred speech. MRI brain scans showed extensive white matter disease that was felt to be less confluent than that usually seen in a leukodystrophy and less extensive than CADASIL. Further testing showed normal evoked potentials and nerve conduction studies and CSF analysis was negative for oligoclonal bands. 2ii declined rapidly, lost all speech and died at 40years from bronchopneumonia. Post- mortem examination of her brain is described below.

**Figure 2:**
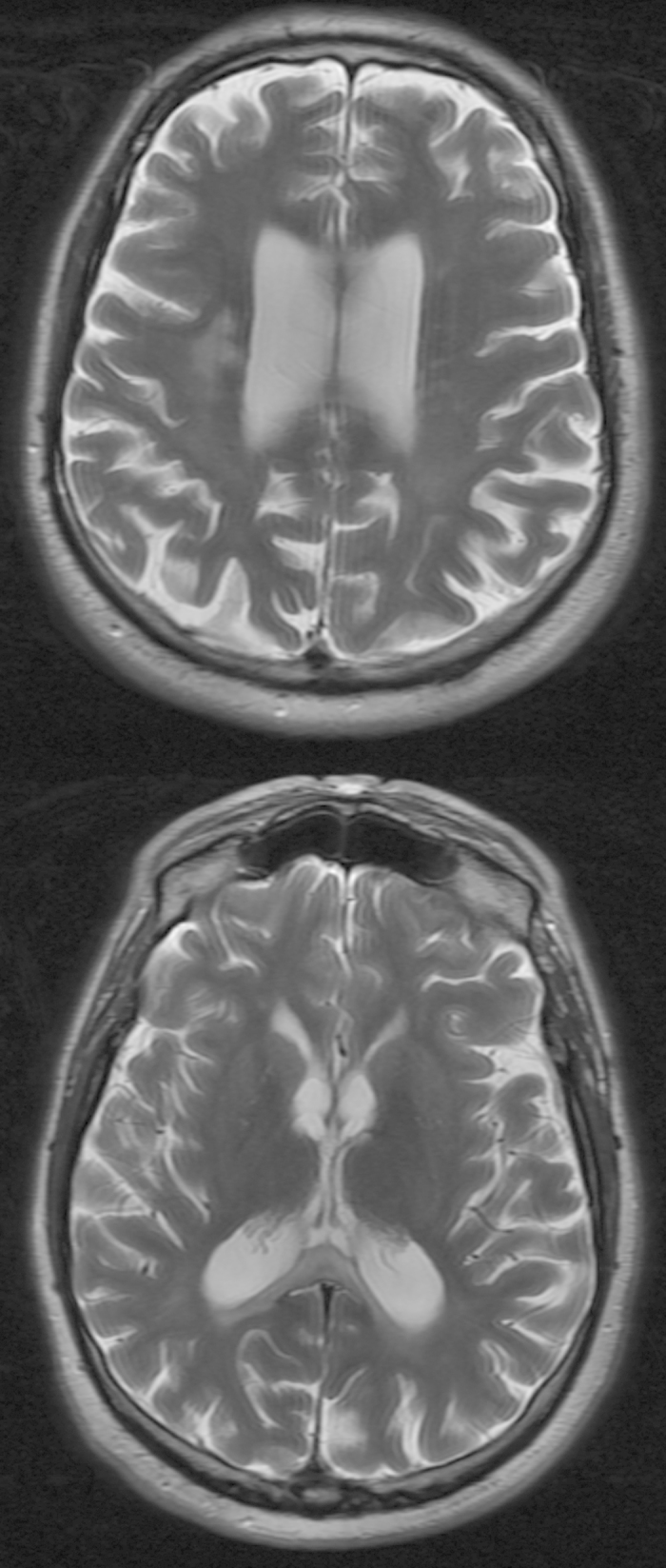
Axial T2-weighted images including the corpus callosum and peri-rolandic white matter. The MRI shows multifocal involvement of the corpus callosum with volume loss, particularly posteriorly. There is asymmetric white matter involvement with sparing of the sub-cortical u-fibres. White matter deep to the pre-central and post-central gyri is specifically involved. No signal change is found within the pyramidal tracts more inferiorly. In places there is apparent involvement of white matter lying transverse to the long axis of the lateral ventricles. Otherwise there is mild cerebellar volume loss, mild parietal and frontal atrophy. The brainstem is normal.

2i presented to clinical services at 57years with slurred speech and difficulty finishing sentences. At this time he was complaining of aching limbs, and now found his writing to be clumsy and his walking slower and more unsteady. He had also noted some memory loss and his family felt that his personality had changed. 2i was working as an agricultural contractor at the time and concerns had been raised by a farm manager that he was not safe operating farm machinery and was not performing routine tasks appropriately. MRI brain scanning at this time showed volume loss in the frontal and parietal lobes and thinning of the corpus callosum. There was also patchy abnormal signal in the white matter of the corpus callosum and in both parietal and frontal lobes (Fig. 2). EMG and nerve conduction studies were normal. Examination showed only minimal limb and gait ataxia, but marked ideomotor and constructional apraxia. A Mini-Mental State examination indicated a substantial cognitive impairment with particular deficits in orientation in time and place and ability to repeat phrases and perform simple arithmetic. His condition declined very rapidly with assessment 6 months later revealing almost no speech and limited spontaneous movement with poor sitting balance. He had also become incontinent of urine and was beginning to struggle with fluids. 2i died 2 months later, aged 59 years having suffered several protracted, generalised seizures.

### Genetic analysis

Exome sequencing was undertaken on individual 2i. A mean depth of sequencing coverage of 54X was attained across targeted exome regions. A total of 24,755 variants were called for this individual; the data passed all standard quality checks. A heterozygous non-synonymous c.C2344G variant resulting in p.R782G was observed in exon 18 of the *CSF1R* gene (defined according to RefSeq transcript NM_005211). This variant was confirmed by Sanger sequencing in an NHS CPA accredited laboratory. This variant is not found in dbSNP 138 or in a public repository of exome data from 6500 individuals (http://evs.gs.washington.edu/EVS/) and was designated as novel to this patient.

### Neuropathology

#### Case 2ii Macroscopic examination.

**Figure 3:**
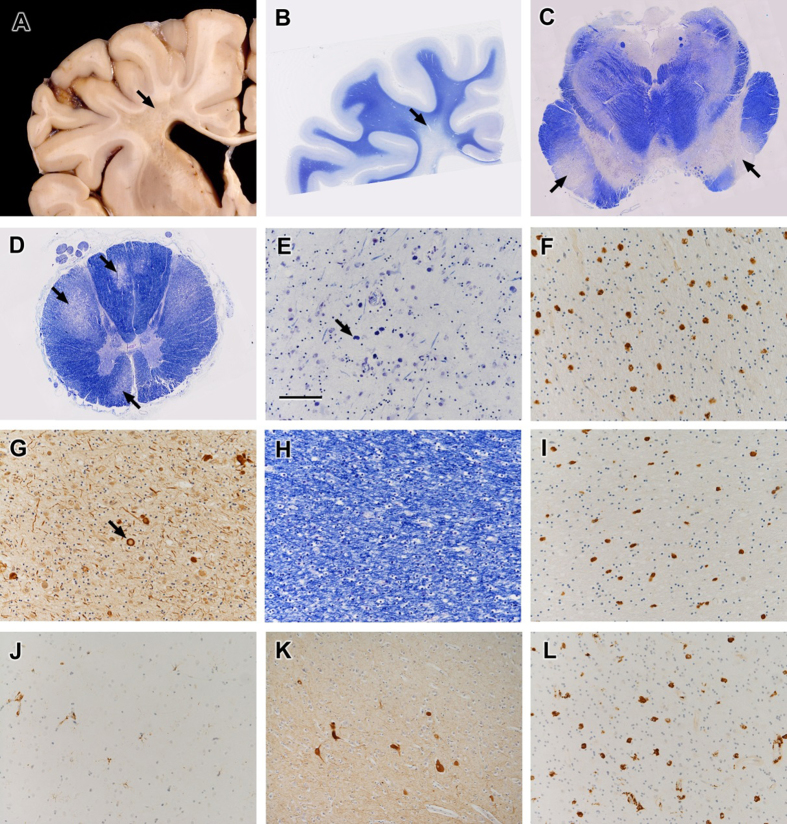
Case 2ii. Post mortem neuropathology. (**a**) Coronal section of cerebrum showing granular degeneration of deep white matter (arrow). (**b**) Low power histology of frontal lobe showing extensive degeneration of deep cerebral white matter (arrow) and corpus callosum with relative sparing of subcortical white matter (LFB/Nissl). Similar degenerative changes affected the corticospinal tracts in the brainstem (**c**. midbrain, arrows) and corticospinal tracts and dorsal columns in the spinal cord (**d**. arrows) (LFB/Nissl). Affected cerebral white matter showed severe depletion of myelinated fibres (**e**. LFB/Nissl) with pigmented macrophages (**e**, arrow and **f**. CD68 immunohistochemistry) and scattered axonal swellings (**g**. arrow, neurofilament immunohistochemistry). Apparently normal subcortical white matter had preserved myelinated fibres (**h**. LFB/Nissl) but still had an abnormal population of macrophages (**i**. CD68 immunohistochemistry). Despite the CSF1R mutation, microglia and perivascular macrophages in the grey matter appeared morphologically normal (**j**. CD68 immunohistochemistry). The deep layers of the cerebral cortex contained morphologically abnormal, swollen neurons (**k**. neurofilament immunohistochemistry). Case 1i. Similar histological features, including a similar distribution of macrophages, were present in affected cerebral white matter in the biopsy sample (**l**. CD68 immunohistochemistry). Figures e-f all taken with x20 objective, scale bar shown in fig e = 100 μm.

External examination of the brain showed mild cerebral atrophy, most prominent in the frontal lobes. Coronal sections showed atrophy of the corpus callosum and patchy discolouration of the centrum semiovale with poorly defined areas of grey granularity around the apices of the lateral ventricles (Fig. 3a). The brainstem was atrophic with the rostro-caudal tracts being prominently affected. The cerebellar hemispheres were atrophied. The spinal cord appeared macroscopically normal.

*Histological examination.* Degenerative changes were present in the white matter involving predominantly cerebral white matter (Fig. 3b), particularly in the centrum semiovale and corpus callosum with relative sparing of subcortical u-fibres, corticospinal tracts in the brainstem (Fig. 3c) and in the spinal cord the crossed and uncrossed corticospinal tracts and the dorsal columns (Fig. 3d). The abnormality consisted of severe loss of myelinated fibres with scattered pigment-containing macrophages (Fig. 3e,f) and occasional axonal spheroids (Fig. 3g). Macrophages were also present in otherwise apparently normal cerebral white matter (Fig. 3h,i). Ramified microglia were largely absent from the affected white matter, but were present with a normal density and morphology in the grey matter (Fig. 3j). There were occasional dysmorphic neurons in the cerebral cortex, (Fig. 3k), particularly in the deeper laminae, but the grey matter was relatively normal elsewhere. Ultrastructural examination revealed lamellar bodies and fingerprint-like profiles in the macrophages within affected areas. At the time, the pathological process was interpreted as a leukodystrophy associated with neuroaxonal dystrophy, with features most consistent with adult pigmentary sudanophilic leukodystrophy.

#### Case 1i

Essentially identical histological features to those described above (Case 2ii), including depletion of myelinated fibres, pigmented macrophages and axonal spheroids, were identified in a biopsy of affected cerebral white matter. A similar pattern of macrophages within the lesion is illustrated (Fig. 3l).

## Discussion

The striking clinical and pathological similarities between the hereditary white matter diseases HDLS and POLD have been noted for several years^11^, but until recently a definitive molecular link between the two disorders had not been made. In 2012 Rademakers *et al.* reported fourteen families with HDLS in whom a combination of linkage analysis and exome sequencing had revealed mutations in the tyrosine kinase domain of *CSF1R*. Functional studies suggested that the mutations affect the kinase activity of the protein, probably altering the phosphorylation of downstream targets. In addition the authors speculated that the mutant CSF1R might assemble into non functional homodimers and wildtype-mutant heterodimers inducing a dominant negative disease mechanism. This gene codes for a cell-surface receptor involved in the regulation of survival, and differentiation of mononuclear phagocytic cells, including microglia in the central nervous system. The key role for CSF1 in maintaining the microglial population has recently been highlighted by the observation that administration of CSF1 antibody can reversibly deplete CNS microglia in rodent models^12^. The discovery of *CSF1R* mutations added HDLS to the emerging class of primary microglial disorders that have been termed microgliopathies. Of particular note, we identified macrophages in otherwise apparently normal cerebral white matter (case 2ii), consistent with a primary role for macrophages/microglia in causing the lesions as indicated by the genetics.

More recently, by sequencing two biopsy proven families, Rademakers’ group have confirmed that mutations in *CSF1R* also underlie POLD. Indeed, not only are the mutations in the same domain of the gene as those found in HDLS families, one mutation was in fact the same as that previously reported with this pathology^8^. The unifying term “adult-onset leukoencephalopathy with axonal spheroids and pigmented glia” has been proposed to encompass this disease spectrum^11^.

We report a family with a novel mutation in *CSF1R* and a particularly rapid disease course with presentation to death occurring within two to three years in all three family members. The mutation that we report changes the same amino acid position as that reported by Kinoshita *et al.*^7^ in an HDLS family and more recently by Nicholson *et al.*^8^ in a POLD family. The mutation reported in both of these previous families changes an arginine to a histidine at position 782 of the protein. This position is strongly conserved between species and within members of the CSF1/platelet derived growth factor family of tyrosine kinase. Previous authors have discussed the likely mechanism of action of HDLS/POLD mutations being a reduction in autophosphorylation of the kinase domain. After binding to the CSF1 ligand, CSF1R is believed to form homodimers before autophosphorylation of its kinase domain. This autophosphorylation precedes CSF1R-dependent phosphorylation of downstream targets, meaning that CSF-1 signaling is critically dependant on this.

We have not performed functional validation for the R782G mutation identified in this family; there is therefore a possibility that this variant is not pathogenic. However, Nicholson *et al.*^8^ showed that CSF1R harbouring the R782H mutation lacks detectable autophosphorylation following activation *in cyto.* In light of this, we consider that it is highly likely that the more severe R782G amino acid change we observe is also pathogenic through the same mechanism. Arginine and histidine would have a similar charge at neutral pH, but substitution to a glycine would increase the negative charge at this locus and this may potentially provide an explanation for the apparently more severe disease course. Addition of a more polar amino acid at position 782 may have a more deleterious effect on this process. It should also be considered that this family may carry an additional mutation or polymorphism elsewhere in the genome that may affect disease progression, or may have been subject to an adverse environmental influence that accounts for the particularly rapid disease course that family members have experienced.

In many individual instances of adult onset leukodystrophy the combination of clinical and MRI brain features are not specific enough for confident clinical diagnoses to be made without further specific testing. In the past families have often had to wait for post mortem findings to understand further which disease process has been involved, and what the broader family risk may be. For some leukodystrophies improvements in MRI scanning and interpretation have allowed the delineation of diagnostic algorithms that pinpoint the likely underlying mechanism and often the specific gene^13^, but for HDLS and POLD, MRI and clinical features have thus far often not been recognised as these specific entities, and a wide variety of ante-mortem diagnoses have been considered^6^,^9^. For family member 2i in this report loss of volume in the corpus callosum, frontal and parietal white matter, together with T2 hyperintensities in the same regions were seen. These changes were apparent at a point in the patients’ disease course at which clinical features were rather non specific (memory loss, personality change and unsteadiness) and would not necessarily have pointed to a white matter disorder. This pattern of changes is certainly in keeping with descriptions of previous patients with HDLS and POLD and it may be that in time a sequence of signature changes will be recognisable. However, as with many areas of adult and paediatric neurology in which a great variety of different genes can underlie very similar clinical and radiological presentations, molecular genetics may provide the fastest route forward. It is likely that there will be increasing use of rapid, panel-based, DNA testing for diagnosing disorders that lack specific clinical signatures, or for which, as yet, the clinical signature has not been elucidated. The ability to provide antemortem diagnoses for individuals and families in such circumstances will improve the prognostic information that can be provided, and in the future may allow more specific treatments. In addition, a molecular result will allow for clarification of the broader family risk where this is desired, and for predictive genetic testing in younger members.

## Methods

### Genetic analysis

DNA was available for individual 2i, isolated from peripheral blood lymphocytes. Exome enrichment was performed using the SureSelect Human All Exon V5.0 kit (Agilent), prior to sequencing on the Illumina HiSeq 2000 system. Following sequencing, reads were aligned to reference genome GRCh37 (hg19) using NovoAlign v2.08.02 (http://www.novocraft.com/products/novoalign/), with duplicate reads flagged using Picard (http://broadinstitute.github.io/picard/). Depth of coverage for target regions was assessed using BEDTools v2.17 14. Following alignment, variants were called using SAMtools v0.1.18 15.

Called variants were annotated with functional effect predictions and population allele-frequencies using ANNOVAR v2013Feb21 16. Sample provenance was ensured through independent application of a SNP based identity panel 17. We performed a tiered analysis of quality filtered variant data, interrogating strong candidate genes in the first instance (namely *CSF1R* and *LMNB1*).

No historical DNA samples of sufficient quality were available from family members 1i or 2ii to look for the p.R782G CSF1R change.

### Neuropathology

The formalin fixed brain and spinal cord from 2ii were examined macroscopically and sampled extensively for histology. Sections were stained with haematoxylin & eosin and luxol fast blue/Nissl and selected sections were immunostained for neurofilament, PGM1, GFAP and ubiquitin. A similar analysis was performed on the brain biopsy from 1i.

## Author Contributions

N.F. wrote the main manuscript text and prepared Fig. 1. Figure 2 together with its legend was prepared by A.D. Figure 3 together with its legend and the pathology methods and results was prepared by J.N. The genetic analysis methods and results were prepared by R.P. and S.E. Clinical care and phenotyping was undertaken by N.F., S.H. and S.B. Molecular genetic analysis and interpretation was undertaken by R.P. and S.E. Radiology undertaken by A.D. Pathological analysis was undertaken by J.N. and D.E.

## Additional Information

**How to cite this article**: Foulds, N. *et al*. Adult-Onset Leukoencephalopathy with Axonal Spheroids and Pigmented Glia Caused by a Novel R782G Mutation in *CSF1R*. *Sci. Rep.* **5**, 10042; doi: 10.1038/srep10042 (2015).
